# Taï Forest Virus Does Not Cause Lethal Disease in Ferrets

**DOI:** 10.3390/microorganisms9020213

**Published:** 2021-01-21

**Authors:** Zachary Schiffman, Feihu Yan, Shihua He, Kevin Tierney, Wenjun Zhu, Karla Emeterio, Huajun Zhang, Logan Banadyga, Xiangguo Qiu

**Affiliations:** 1 Department of Medical Microbiology and Infectious Diseases, University of Manitoba, Winnipeg, MB R3E 0J9, Canada; zachary.schiffman@canada.ca (Z.S.); karla.emeterio@canada.ca (K.E.); huajun_zhang@126.com (H.Z.); 2 Special Pathogens Program, National Microbiology Laboratory, Public Health Agency of Canada, Winnipeg, MB R3E 3R2, Canada; shihua.he@canada.ca (S.H.); kevin.tierney@canada.ca (K.T.); wenjun.zhu@canada.ca (W.Z.); xiangguo.qiu@canada.ca (X.Q.); 3 Key Laboratory of Jilin Province for Zoonosis Prevention and Control, Changchun Veterinary Research Institute, Chinese Academy of Agricultural Sciences, Changchun 130122, China; yanfh1900@gmail.com

**Keywords:** filovirus, ebolavirus, Côte d’Ivoire ebolavirus, Taï Forest virus, TAFV, pathogenesis, ferret, animal model, viral hemorrhagic fever

## Abstract

Filoviruses are zoonotic, negative-sense RNA viruses, most of which are capable of causing severe disease in humans and nonhuman primates, often with high case fatality rates. Among these viruses, those belonging to the *Ebolavirus* genus—particularly Ebola virus, Sudan virus, and Bundibugyo virus—represent some of the most pathogenic to humans. Taï Forest virus (TAFV) is thought to be among the least pathogenic ebolaviruses; however, only a single non-fatal case has been documented in humans, in 1994. With the recent success of the ferret as a lethal model for a number of ebolaviruses, we set out to evaluate its suitability as a model for TAFV. Our results demonstrate that, unlike other ebolaviruses, TAFV infection in ferrets does not result in lethal disease. None of the intramuscularly inoculated animals demonstrated any overt signs of disease, whereas the intranasally inoculated animals exhibited mild to moderate weight loss during the early stage of infection but recovered quickly. Low levels of viral RNA were detected in the blood and tissues of several animals, particularly the intranasally inoculated animals, and all animals mounted a humoral immune response, with high titers of GP-specific IgG detectable as early as 14 days post-infection. These data provide additional insight into the pathogenesis of TAFV.

## 1. Introduction

Filoviruses are zoonotic, negative-sense, single-stranded RNA viruses belonging to the family *Filoviridae* of the order *Mononegavirales*. These viruses are the causative agent of Filovirus Disease (FVD), a form of viral hemorrhagic fever affecting both humans and nonhuman primates (NHPs), often with high case fatality rates [1,2]. Although the family *Filoviridae* encompasses a total of six distinct genera, only members of the *Ebolavirus* and *Marburgvirus* genera are known to be capable of causing disease in humans [3]. Among the ebolaviruses, Ebola virus (EBOV), Sudan virus (SUDV), and Bundibugyo virus (BDBV) are considered the most pathogenic, having been responsible for a number of sporadic outbreaks since 1976, with overall case fatality rates ranging from 33% to 53%, depending on the virus [3]. In contrast, Reston virus (RESTV) does not cause disease in humans but does cause serious, often lethal disease in NHPs, specifically cynomolgus macaques (*Macaca fascicularis*) [4]. Although studies have demonstrated that the glycoprotein (GP) of the recently identified Bombali virus (BOMV) is capable of mediating entry into human cells, it is not yet known whether BOMV can cause disease in humans [5,6,7]. Lastly, there has been only a single reported case of Taï Forest virus (TAFV) in humans, which occurred in 1994 in Côte-d’Ivoire when a 34-year-old veterinarian became ill after performing an autopsy on a wild chimpanzee found in the Taï Forest [8,9,10]. The patient initially presented with non-specific symptoms including fever, nausea, vomiting, diarrhea, rash, and abdominal pain that were later linked to TAFV, a novel ebolavirus. The patient ultimately made a full recovery, and since then no additional human cases of TAFV infection have been reported [8].

Animal models represent a critical tool for studying filovirus pathogenesis, with a number of models, including immunocompetent and immunocompromised mice, guinea pigs, hamsters, and NHPs, having been developed for most ebolaviruses [11,12,13,14,15,16]. Although TAFV infections have previously been evaluated in NHPs (*Macaca fascicularis*), immunodeficient mice (IFN-α/βR^−/−^), and Egyptian fruit bats (*Rousettus Aegypticus*) [15], there still remains no well-characterized animal model for studying TAFV pathogenesis. This is likely due to the fact that TAFV has yet to cause any significant outbreaks, and by extension can be regarded as a low public health risk and therefore a low research priority.

The domestic ferret (*Mustela putorius furo*) has become increasingly popular as a small animal model for evaluating filovirus transmission and pathogenesis, in addition to efficacy of candidate countermeasures [17]. The main advantage of the ferret as a model for filoviruses is that, unlike the majority of rodent models, ferrets are susceptible to most wild type filoviruses without the need for host adaptation, and they exhibit disease progression that closely parallels that observed in humans and NHPs [17]. Interestingly, infection in ferrets with EBOV [18,19], SUDV [20], BDBV [19], and RESTV [21] results in uniform lethality, whereas infection with marburgviruses, namely Marburg virus (MARV) and Ravn virus (RAVV), does not result in disease [22,23].

Due to the lack of available animal models for TAFV, and the success of the ferret as a lethal model for a number of ebolaviruses, we set out to evaluate the suitability of ferrets as a small animal model for evaluating TAFV pathogenesis. A well-characterized animal model for TAFV could provide additional insight into the pathogenesis of TAFV and, by extension, ebolaviruses and filoviruses in general. Moreover, having an animal model for TAFV would also prove useful for the evaluation of cross-reactive or pan-filovirus therapeutics and vaccines.

## 2. Materials and Methods

### 2.1. Ethics Statement

All animal work involving infectious TAFV was performed in the containment level-4 (CL-4) facility at the Canadian Science Center for Human and Animal Health (CSCHAH) of the Public Health Agency of Canada in Winnipeg, Canada. All experiments were approved by the Animal Care Committee of the CSCHAH (Animal Use Document# H16-026, approved 10 December 2019) in accordance with guidelines from the Canadian Council on Animal Care. Animals were acclimatized for 7 days prior to infection, given food and water ad libitum, and monitored twice daily. Environmental enrichment was provided throughout the study.

### 2.2. Viruses

Taï Forest virus (TAFV) isolate Tai Forest virus/H.sapiens-tc/CIV/1994/Tai Forest-CDC807212 (GenBank: KU182910.1) was previously obtained from the Centers for Disease Control and Prevention (Atlanta, GA, USA) with an unknown passage history. The virus used in this study was a P4 working stock previously passaged on Vero E6 cells at CSCHAH. Deep sequencing of the P4 working stock revealed the virus was 100% identical to the TAFV reference sequence (GenBank: KU182910.1). The stock virus was tested for mycoplasma contamination using the Microstart RESEARCH Mycoplasma Detection kit as per the manufacturer’s instructions (Sartorius, Goettingen, Germany).

### 2.3. Animals

Male (*n* = 4) and female (*n* = 4) ferrets (*Mustela putorius furo*), ~14–16 weeks old and weighing approximately 0.6–0.75 kg, were purchased commercially from Marshall Bioresources (New York, NY, USA). All animals were given anticoccidials, in addition to vaccines against distemper and rabies virus at time of purchase. Additionally, all animals were implanted with an IPTT-300 temperature and ID transponder (BioMedic Data Systems Inc., Seaford, DE, USA). All animals were housed in the CL-4 facility at CSCHAH within negative pressure caging units supplied with high-efficiency particulate air (HEPA)-filtered air.

**Figure 1:**
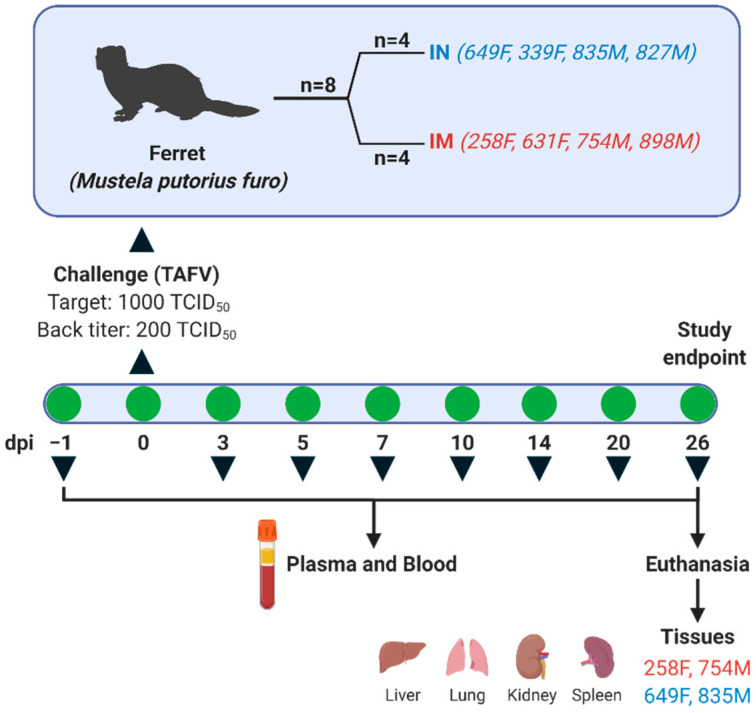
Experimental Design. Ferrets were divided into two groups (*n* = 4/group) containing equal numbers of males and females. Animals were challenged intramuscularly (IM) or intranasally (IN) with Taï Forest virus (TAFV) at a target dose of 1000 TCID_50_. Whole blood and plasma were collected on −1, 3, 5, 7, 10, 14, 20, and 26 days-post infection (dpi) for subsequent viral RNA, hematology, complete blood count, and IgG analysis. Tissues (liver, lung, spleen, and kidney) were harvested at study end-point from animals 258F, 754M, 649F, and 835M for viral RNA analysis.

Ferrets were randomly assigned into two groups (*n* = 4), each with an equal number of male and females, and challenged either intramuscularly (IM) or intranasally (IN) with TAFV at a target dose of 1000 TCID_50_ (back titered to 200 TCID_50_) (Figure 1), diluted in plain Dulbecco’s Modified Eagle Medium (DMEM) High Glucose (Hyclone, Lafayette, CO, USA) to a final volume of 500 μL. The inoculum was equally administered (250 μL/site) at two sites (both rear quadriceps for IM and both nostrils for IN) to ensure consistent infection among animals. Animals were monitored daily for signs of disease, which included changes in body weight, temperature, physical activity, and food/water intake. Whole blood was collected at −1, 3, 5, 7, 10, 14, and 20 days post-infection (dpi), and at the study endpoint on day 26 post-infection to evaluate plasma biochemistry and complete blood count (CBC). Tissues, including liver, spleen, kidney, and lung, were harvested at study endpoint (26 dpi) from one male and one female from each group (258F, 754M, 649F, and 835M) for downstream viral RNA analysis.

### 2.4. Plasma Biochemistry and Complete Blood Counts

Whole blood was collected in lithium heparin tubes and Ethylenediaminetetraacetic acid (EDTA) tubes (BD, Franklin Lakes, NJ, USA) for subsequent plasma biochemistry and CBC analysis, respectively. Plasma biochemistry and CBC were evaluated using the VetScan VS2 blood analyzer and VetScan HM5 hematology system, respectively (Abaxis, Union City, CA, USA), per the manufacturer’s instructions.

### 2.5. Quantification of Viral RNA by RT-qPCR

Total RNA was extracted from blood using the QIamp viral RNA minikit (Qiagen, Germantown, MD, USA), per the manufacturer’s protocol. Total RNA was extracted from 30 mg homogenized tissue using the RNeasy Kit, according to the manufacturer’s instructions (Qiagen, Germantown, MD, USA). Viral RNA levels were determined via reverse transcription quantitative PCR (RT-qPCR) using the Lightcycler 480 RNA Master Hydrolysis probes kit (Roche, Indianapolis, IN, USA) with two sets of primers and probes targeting either TAFV L or NP (Table S1). All RT-qPCR reactions were performed using a Quantstudio 3 Real-time PCR system (Applied Biosystems, Foster City, CA, USA) with the following cycling conditions: 63 °C for 3 min, 95 °C for 30 s, followed by 45 cycles of 95 °C for 15 s, 60 °C for 30 s. Samples with cycle threshold (CT) values of ≤36 were considered positive, whereas samples with CT values >36 but <40 were considered equivocally positive. Any sample with a CT ≥40 was considered negative [24].

### 2.6. Generation and Purification of TAFV GP

A plasmid encoding human codon optimized TAFV GP1 possessing an S tag was transfected into Expi293 cells using the ExpiFectamine 293 Reagent (Life Technologies, Eugene, OR, USA), according to the manufacturer’s instructions. Cells were harvested four days post-transfection by centrifugation at 3000× *g* for 10 min. EDTA was added to the supernatant to a final concentration of 10 mM, and the supernatant was subsequently incubated with S-protein agarose beads (Novagen) overnight at 4 °C with gentle shaking. The beads were collected in a spin filter column with gravity flow. The column was washed three times with wash buffer (0.15 M NaCl, 20 mM Tris-HCl, 0.1% TritonX-100, pH 7.5) by centrifuging at 500× *g* for 5 min and eluted with 0.1 M citric acid (pH 2.5). The eluate was immediately neutralized with 3 M Tris base, followed by dialysis against PBS. Protein was then concentrated using Amicon Ultra-15 centrifugal filter units, and the purified proteins quantified using the BCA protein assay kit (Thermo Fisher Scientific, Waltham, MA, USA), per the manufacturer’s instructions.

### 2.7. Evaluation of Humoral Immune Response by ELISA

Plasma samples collected on −1, 14, 20, and 26 dpi were subjected to an enzyme-linked immunosorbent assay (ELISA) developed in-house to quantify TAFV GP-specific immunoglobulin G (IgG). Briefly, 96-well half-area high binding plates (Corning, NY, USA) were coated overnight at 4 °C with 1 μg/mL TAFV GP (described above) diluted in 50 mM carbonate buffer (pH 9.6). The following day, plates were blocked for 1 h at 37 °C with 5% (*w*/*v*) skim milk (BD, Franklin Lakes, NJ, USA) diluted in phosphate-buffered saline (PBS) containing 1% (*v*/*v*) Tween-20 (1% PBST). Plasma samples were serially diluted 10-fold in 1% (*w*/*v*) bovine-serum albumin (BSA) prepared in PBS (1% BSA) starting at a dilution of 1:400 for all samples except pre-bleed (−1 dpi), for which the initial dilution was 1:100. After blocking, the plates were gently blotted, 30 μL diluted plasma was added to wells in triplicate, and plates were incubated for 1 h at 37 °C. Plates were then washed 4 times with 150 μL wash buffer (0.1% PBST) using a microplate washer. After washing, plates were blotted to remove residual wash buffer and subsequently incubated for 1 h at 37 °C with 30 μL/well HRP-conjugated goat anti-ferret IgG secondary antibody (Bethyl laboratories, Montgomery, TX, USA) diluted 1:10,000 in 1% BSA. Plates were then washed as described and incubated in the dark with 50 μL/well TMB (Thermo Fisher Scientific, Waltham, MA, USA) for 30 min at room temperature. The optical density (OD) was recorded at 650 nm using the Synergy HTX plate reader (Bio-Tek, Winooski, VT, USA). The OD cut-off was defined as the average of the pre-bleeds (1/100 dilution) plus 3 standard deviations.

### 2.8. Data Analysis

Interpretation of all RT-qPCR data was undertaken using the Quantstudio Design and Analysis Software (Applied Biosystems, Foster City, CA, USA). All graphs and statistics were generated using GraphPad Prism 8.0. All illustrations were prepared using Biorender (BioRender.com). Statistical analysis for blood biochemistry and complete blood count were performed using a two-way ANOVA analysis with Tukey’s multiple comparisons test, with individual variances computed for each comparison. *p* values are as follows >0.05 (ns), ≤0.05 (*), ≤0.01 (**), ≤0.001 (***), ≤0.0001 (****).

## 3. Results

### 3.1. Ferrets Did Not Develop Lethal Disease Following TAFV Infection

**Figure 2:**
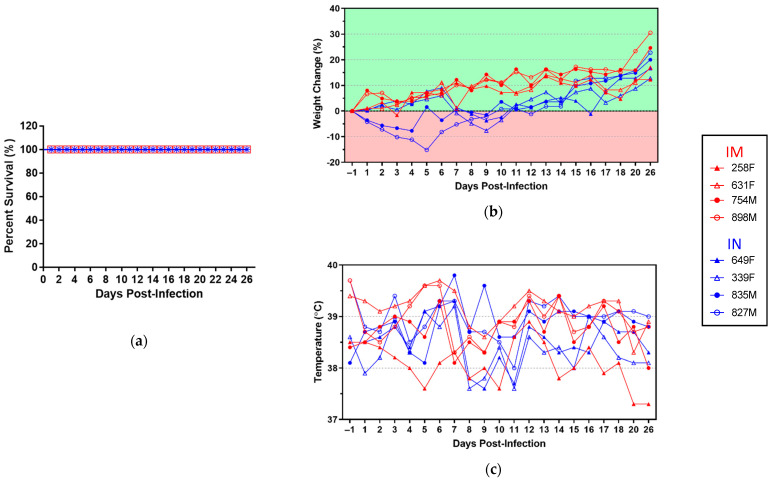
Clinical parameters of TAFV-infected ferrets. Survival curves (**a**), percent weight change (**b**), and temperature (**c**) for each animal over the duration of the study. Individual animal IDs and sex (F/M) are indicated in the key. IM, intramuscular inoculation; IN, intranasal inoculation.

To investigate the susceptibility of ferrets to infection with TAFV, animals were divided into two groups with equal numbers of males and females and infected either IM or IN with 200 TCID_50_ TAFV (Figure 1). All animals survived challenge with TAFV regardless of inoculation route (Figure 2a). Interestingly, however, all animals within the IN, but not IM, group demonstrated mild to moderate weight loss at some point prior to 9 dpi, with weight loss most pronounced in animals 835M and 827M at 4 and 5 dpi, respectively (Figure 2b). None of the animals in either group exhibited body temperature perturbations outside of the normal range (37.8–40 °C [25]) (Figure 2c), nor did any of the animals demonstrate reduced physical activity or food/water intake throughout the duration of the study.

### 3.2. Transient Viremia Early after Infection

To evaluate viral RNA levels from the blood, total RNA samples were subjected to RT-qPCR using two primer/probe sets targeting either TAFV nucleoprotein (NP) or polymerase (L). All animals challenged via the IN route were weakly positive (CT < 36) for NP and/or L at 7 dpi (Table 1). In contrast, only two animals within the IM group were weakly positive, namely 898M at 5 dpi for NP and 631F at 7 dpi for L (Table 1). Several blood samples from both the IM and IN groups were equivocally positive at 3, 7, and 10 dpi (CT > 36 but <40), potentially indicative of extremely low levels of viral RNA. None of the animals had detectable viral RNA in blood at 3, 14, 20, and 26 dpi (Table 1). At study endpoint (26 dpi), tissues from four animals (two each from the IM and IN groups) were also assessed for viral RNA using the same RT-qPCR methodology (Table 2). The spleen was uniformly positive or equivocally positive for viral RNA in all four animals, whereas the liver and kidney were uniformly negative. The lung samples were similarly negative, except for the sample from 649F, which was considered to be equivocally positive.

### 3.3. No Major Perturbations in Hematology or Complete Blood Counts

**Figure 3:**
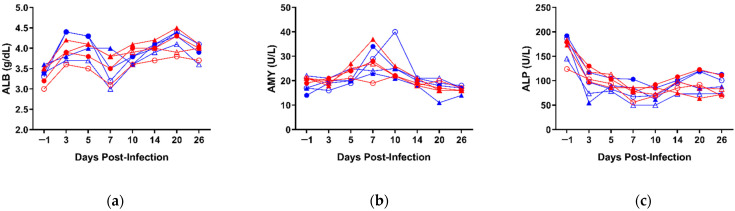

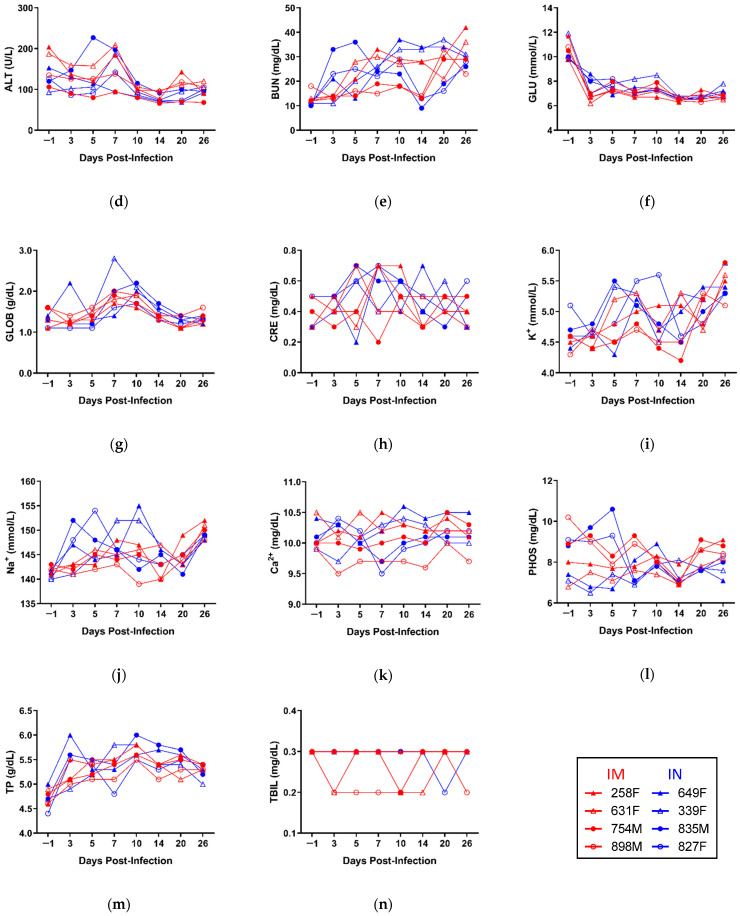
Biochemical parameters of TAFV-infected ferrets. Whole blood was collected from each animal on days −1, 3, 5, 7, 10, 14, 20, and 26 post-infection, and the concentrations of the following analytes measured: (**a**) albumin (ALB); (**b**) amylase (AMY); (**c**) alkaline phosphatase (ALP); (**d**) alanine aminotransferase (ALT); (**e**) blood urea nitrogen (BUN); (**f**) blood glucose (GLU); (**g**) globulin (GLOB); (**h**) creatinine (CRE); (**i**) potassium (K^+^); (**j**) sodium (Na^+^); (**k**) calcium (Ca^2+^); (**l**) phosphorus (PHOS); (**m**) total protein (TP); (**n**) total bilirubin (TBIL). Individual animal IDs and sex (F/M) are indicated in the key. IM, intramuscular inoculation; IN, intranasal inoculation.

Analysis of blood biochemistry and cell counts was performed at all time-points to further characterize TAFV pathogenesis and the impact on both the circulatory and immune systems. All animals displayed relatively normal blood biochemistries, with values for all parameters within normal physiological ranges (Figure 3 and Figure S1). Overall, none of the biochemical parameters evaluated were statistically different between the two groups, with the exception of glucose (GLU), which was significantly lower at 3 dpi in the IM group compared to the IN group (Figure S1).

**Figure 4:**
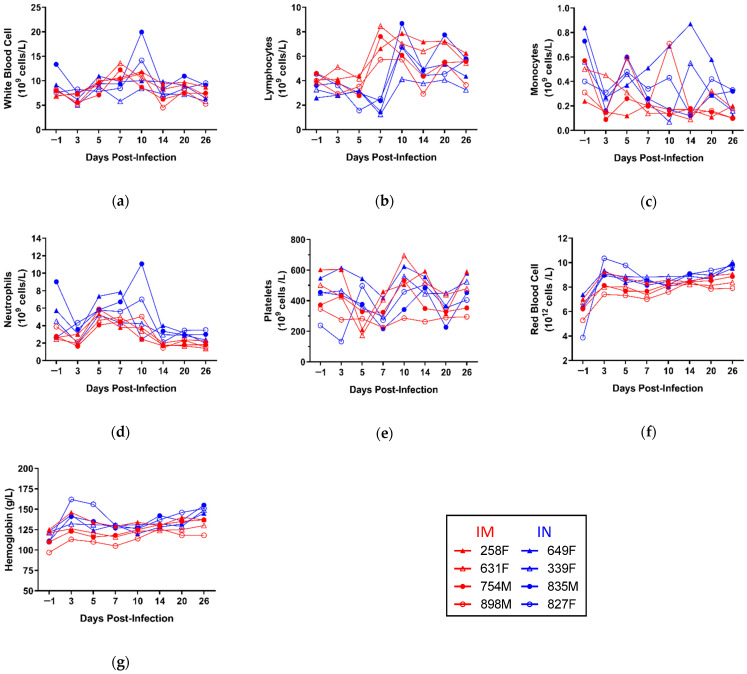
Complete blood counts of TAFV-infected ferrets. Whole blood was collected from each animal on days −1, 3, 5, 7, 10, 14, 20, and 26 post-infection and the concentrations of the following analytes measured: (**a**) white blood cells (WBC); (**b**) lymphocytes (LYM); (**c**) monocytes (MON); (**d**) neutrophils (NEU); (**e**) platelets (PLT); (**f**) red blood cells (RBC) and (**g**) hemoglobin (HB). Individual animal IDs and sex (F/M) are indicated in the key. IM, intramuscular inoculation; IN, intranasal inoculation.

Analysis of blood cell counts also revealed no major perturbations, although a few notable trends were observed (Figure 4). Animal 835M was found to have elevated white blood cells (WBC) at 10 dpi, reflecting an increase in both lymphocytes (LYM) and neutrophils (NEU) at the same time point, and animal 649F showed a distinct peak in monocytes (MON) at 14 dpi. Although no significant differences were observed between the IM and IN groups for many of the parameters measured, a significant decrease in lymphocytes (LYM) was observed in the IN group at 7 dpi, suggesting possible lymphopenia (Figure S2). Moreover, animals within the IM group demonstrated overall lower MON and NEU counts compared to those within the IN group, although the differences were not statistically significant (Figure S2). Red blood cell (RBC) counts were significantly lower at 3 and 26 dpi, as was hemoglobin (HB) at 26 dpi, in the IM group compared to the IN group (Figure S2).

### 3.4. High Titers of TAFV GP-Specific IgG Detectable as Early as 14 dpi

**Figure 5:**
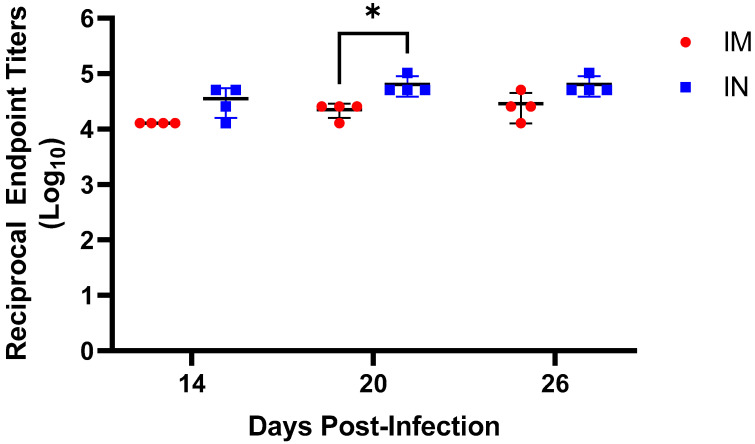
Endpoint titers of TAFV GP-specific IgG. Plasma samples were collected from each animal at 14, 20, and 26 dpi and subjected to ELISA to quantify total amounts of circulating TAFV GP-specific IgG. Values reported represent lowest reciprocal dilution (Log_10_) above the cut-off. IM, intramuscular inoculation; IN, intranasal inoculation. * indicates a *p*-value ≤ 0.05.

To evaluate the humoral immune response mounted after challenge with TAFV, plasma samples collected at −1, 14, 20, and 26 dpi were subjected to an indirect ELISA to quantify titers of TAFV GP-specific IgG. In contrast to the pre-challenge (−1 dpi) plasma samples, which were uniformly negative (Figure S3), all post-infection samples exhibited high GP-specific IgG titers, indicating that all animals seroconverted and mounted a humoral immune response against TAFV GP (Figure 5, Table S2). Overall, the mean TAFV GP-specific IgG titers trended higher in IN-challenged animals than in IM-challenged animals, and the difference was significant at 20 dpi (Figure 5). These data suggest that animals infected via the IN route mounted a more pronounced humoral immune response compared to those infected via the IM route.

## 4. Discussion

Although the ferret has long been considered an exceptional model for influenza viruses [26], it has more recently been shown to be an appealing animal model for evaluating ebolavirus pathogenesis. Indeed, the ferret is highly susceptible to lethal infection with EBOV, SUDV, BDBV, and RESTV, and displays many of the clinical hallmarks associated with disease in humans and NHPs [18,19,20,21]. Nevertheless, we demonstrate here that TAFV—another ebolavirus most closely related to BDBV [27]—did not produce lethal disease in ferrets, regardless of whether the animals were challenged via the IM or IN route.

Despite the fact that no animal developed severe disease in response to TAFV inoculation, our data suggest most animals experienced a productive infection that may have led to mild signs of disease. In particular, animals inoculated via the IN route showed more consistent signs of disease than those inoculated via the IM route. All IN-inoculated animals demonstrated mild to moderate weight loss early post-infection, with a few animals (835M, 827M, and 339F) losing as much as ~5–15% their starting weight. Although weight loss is a key indicator of Ebola virus disease (EVD) in ferrets, it is typically accompanied by other signs, such as elevated temperature, rash, reduced physical activity and/or changes in food/water intake [19,20,21], none of which were observed at any point in this study. Thus, while the weight loss observed here is thought to be virus-induced, we cannot exclude a role for other factors, such as stress. In contrast to the IN-inoculated animals, however, none of the animals within the IM group exhibited substantial weight loss at any point during the study. These data suggest that, unlike the IM-inoculated animals, the IN-inoculated animals experienced a mild form of disease, and they are consistent with the analysis of viral RNA loads.

Although the CT values were relatively high in general, all animals were weakly positive or equivocally positive for viral RNA in the blood around 5–10 dpi, suggesting limited virus replication and viremia. Moreover, low levels of viral RNA were also detected in some tissues, particularly the spleen, further indicating virus dissemination. Interestingly, only IN-inoculated animals showed uniformly positive RT-qPCR results at 7 dpi, whereas almost all IM-inoculated animals showed equivocally positive results, if any. Indeed, the CT values observed in IN-inoculated animals were more numerous and lower in magnitude than those from IM-inoculated animals, suggesting a higher viral burden in the former group.

Despite limited weight loss and low levels of viral replication, none of the animals in either group demonstrated any major perturbations in blood biochemistry outside of their normal range, indicating that little damage was sustained by organs, such as the liver and spleen. Blood cells counts were similarly unremarkable, with the exception of the lymphocyte count. IN-inoculated animals demonstrated significantly lower lymphocyte counts at 7 dpi when compared to IM challenged animals, indicating possible lymphopenia, a common feature of EVD [2]. Although it is difficult to draw any concrete conclusions from these data, it is once again interesting to note that the apparent lymphopenia in the IN-inoculated animals is consistent with their slightly higher viral burden and increased weight loss.

As expected, all animals were found to have high titers of TAFV GP-specific IgG as early as 14 dpi, regardless of inoculation route, indicating that all animals mounted a humoral immune response. Nevertheless, the mean endpoint titers were higher among IN-inoculated animals than among IM-inoculated, suggesting that animals infected via the IN route may have elicited a more pronounced humoral immune response. In the future, it would be interesting to further characterize both the humoral and cell-mediated immune responses to TAFV infection in an effort to gain further insight into whether the absence of lethal disease is the result of a robust immune response. Unfortunately, the lack of commercially available reagents, particularly for immunology, makes such a characterization difficult. In addition, with such high titers of TAFV GP-specific IgG, it would also be interesting to evaluate whether these antibodies exhibit any neutralizing activity, because high neutralizing titers could be a hallmark feature of TAFV infections and critical for preventing lethal disease in both humans and animal models.

Taken together, our findings indicate that although ferrets inoculated with TAFV did not succumb to the virus, they appeared to develop a productive infection with mild signs of disease and a robust immune response, particularly when inoculated via the IN route. These results stand in contrast to those obtained with EBOV, SUDV, BDBV, and RESTV, which cause a serious and uniformly lethal disease in ferrets [19,20,21]. It is unclear why TAFV did not result in lethal disease, although it could be related to the overall pathogenicity of the virus, which is assumed to be low, based on a single non-fatal case in humans and limited experiments in mice and NHPs. Indeed, despite being partially susceptible to EBOV and SUDV, immunodeficient mice (IFN-α/βR^−/−^) are resistant to disease caused by TAFV [28]. Similarly, TAFV was shown to be only partially lethal in cynomolgus macaques, with 3/5 (60%) animals succumbing to disease at 12–14 dpi following inoculation with 1000 PFU [29]. It is worth nothing, however, that MARV and RAVV, which both cause lethal disease in humans and NHPs [30], do not cause disease in ferrets [22,23], similar to TAFV. Conversely, RESTV, which does not cause lethal disease in humans, does so in ferrets [21]. Thus, filovirus pathogenesis may be more complicated than can be accounted for in the ferret model, and more work is required to understand the degree to which these animals do (or do not) recapitulate filovirus disease processes. An important caveat to this interpretation—and a limitation to our study—is that back titration of our TAFV inoculum revealed a dose of 200 TCID_50_, rather than the target dose of 1000 TCID_50_. Although doses of 200 TCID_50_ or less proved lethal for EBOV [19,31] and BDBV [19] in ferrets, it cannot be ruled out that a higher dose of TAFV would result in more serious disease and/or lethality in this animal.

Importantly, following the completion of this study, mycoplasma DNA was detected in our stock preparation of TAFV, suggesting mycoplasma contamination. Nevertheless, we did not observe any signs of respiratory disease or pathology that could be related to mycoplasma infection in the ferrets involved in this study, and we find it unlikely that this mycoplasma contamination could influence the pathogenicity of TAFV in these animals.

The ferret remains a relatively new animal model for studying filovirus pathogenesis and its translatability to humans is unclear. Not only does this work to establish a ferret model of TAFV add to the complexity of our understanding of this model system, but it may also help shed some light on a relatively understudied filovirus. Because it is likely that TAFV continues to circulate in an unknown reservoir host, another human spillover event—or potentially an outbreak—remains a consistent threat [15]. This threat, in addition to our relative ignorance of the virus’s biology, further emphasizes the need for continual research on TAFV.

## Figures and Tables

**Table 1:** Viral RNA load in blood of ferrets infected with TAFV.

Intramuscular (IM).				Intranasal (IN)			
Animal ID	Day Post-Infection	CT		Animal ID	Day Post-Infection	CT	
		L	NP			L	NP
258F	3	-	-	339F	3	-	-
	5	(36.90)	-		5	-	-
	7	-	(38.93)		7	35.48	35.80
	10	-	-		10	-	-
	14	-	-		14	-	-
	20	-	-		20	-	-
	26	-	-		26	-	-
631F	3	-	-	649F	3	-	-
	5	(38.61)	(36.18)		5	(36.06)	(36.91)
	7	35.30	(37.42)		7	35.57	35.34
	10	-	-		10	(37.24)	(39.04)
	14	-	-		14	-	-
	20	-	-		20	-	-
	26	-	-		26	-	-
898M	3	-	-	827M	3	-	-
	5	(39.71)	34.94		5	-	-
	7	-	(37.00)		7	35.62	34.78
	10	-	-		10	-	-
	14	-	-		14	-	-
	20	-	-		20	-	-
	26	-	-		26	-	-
754M	3	-	-	835M	3	-	-
	5	(39.88)	(37.04)		5	-	(39.28)
	7	-	-		7	(36.40)	34.24
	10	-	-		10	-	-
	14	-	-		14	-	-
	20	-	-		20	-	-
	26	-	-		26	-	-

Positive samples (CT < 36) denoted in bold letters, equivocally positive samples (CT > 36, <40) denoted in parentheses, and negative samples (CT ≥ 40) denoted by “-”. CT, cycle threshold; L, target L gene of TAFV; NP, target nucleoprotein gene of TAFV.

**Table 2:** Viral RNA load in tissues of ferrets infected with TAFV.

Route	Animal ID	Tissue	CT	
			L	NP
Intramuscular (IM)	258F	Liver	-	-
		Lung	-	-
		Spleen	35.98	(38.25)
		Kidney	-	-
	754M	Liver	-	-
		Lung	-	-
		Spleen	(37.70)	-
		Kidney	-	-
Intranasal (IN)	649F	Liver	-	-
		Lung	(37.75)	(38.71)
		Spleen	33.47	34.70
		Kidney	-	-
	835M	Liver	-	-
		Lung	-	-
		Spleen	(36.17)	(36.48)
		Kidney	-	-

Positive samples (CT < 36) denoted in bold letters, equivocally positive samples (CT > 36, <40) denoted in parentheses, and negative samples (CT ≥ 40) denoted by “-”. CT, cycle threshold; L, target L gene of TAFV; NP, target nucleoprotein gene of TAFV.

## Data Availability

Data is contained within the article or supplementary material.

## Supplementary Materials

The following are available online at https://www.mdpi.com/2076-2607/9/2/213/s1, Figure S1: Average biochemical parameters of TAFV-infected ferrets, Figure S2: Average complete blood counts of TAFV-infected ferrets, Figure S3: Detection of TAFV GP-specific IgG by ELISA, Table S1: RT-qPCR primer/probe sequences used for the detection of TAFV RNA, Table S2: Endpoint Titers of TAFV GP-specific IgG.
