# Supplementary material for: Taï Forest Virus Does Not Cause Lethal Disease in Ferrets
Source: Microorganisms. 2021 Jan 21;9(2):213. doi: 10.3390/microorganisms9020213 (PMC7909818; doi:10.3390/microorganisms9020213)
Supplement: Supplementary file 1 [file microorganisms-09-00213-s001.pdf]

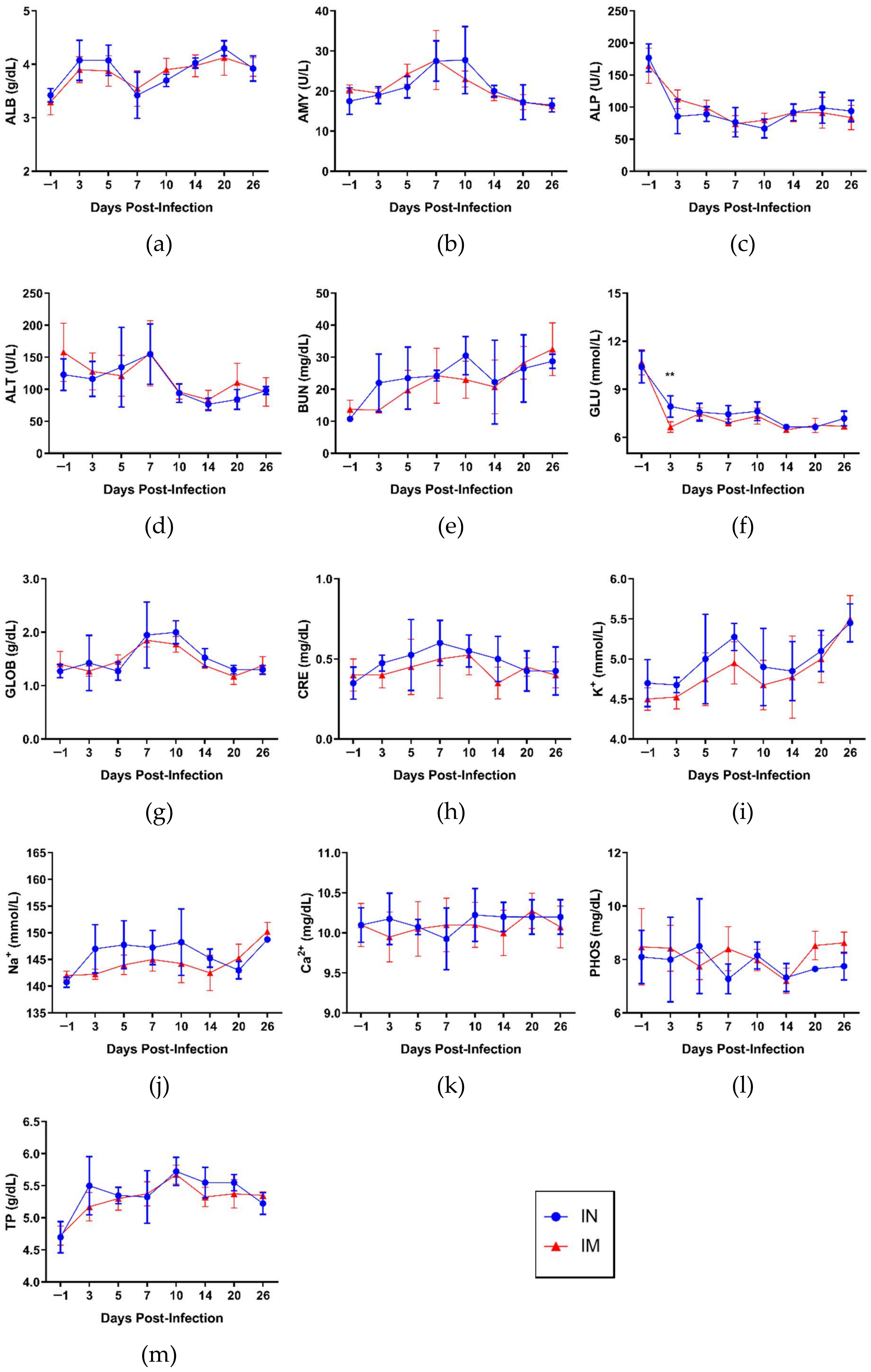
ALB (g/dL)
AMY (U/L)
ALP (U/L)
ALT (U/L)
BUN (mg/dL)
GLU (mmol/L)
**
GLOB (g/dL)
CRE (mg/dL)
K^+ (mmol/L)
Na^+ (mmol/L)
Ca^{2+} (mg/dL)
PHOS (mg/dL)
TP (g/dL)
Days Post-Infection
−1
3
5
7
10
14
20
26
IN
IM
(a)
(b)
(c)
(d)
(e)
(f)
(g)
(h)
(i)
(j)
(k)
(l)
(m)

Figure S1. Average biochemical parameters of TAFV-infected ferrets. Whole blood was collected from each animal on days −1, 3, 5, 7, 10, 14, 20 and 26 post infection and the concentrations of the following parameters measured: (**a**) albumin (ALB); (**b**) amylase (AMY); (**c**) alkaline phosphatase (ALP); (**d**) alanine aminotransferase (ALT); (**e**) blood urea nitrogen (BUN); (**f**) blood glucose (GLU); (**g**) globulin (GLOB); (**h**) creatinine (CRE); (**i**) potassium (K^+); (**j**) Sodium (Na^+); (**k**) calcium (Ca^{2+}); (**l**) phosphorus (PHOS); (**m**) total protein (TP). IM, intramuscular inoculation; IN, intranasal inoculation.

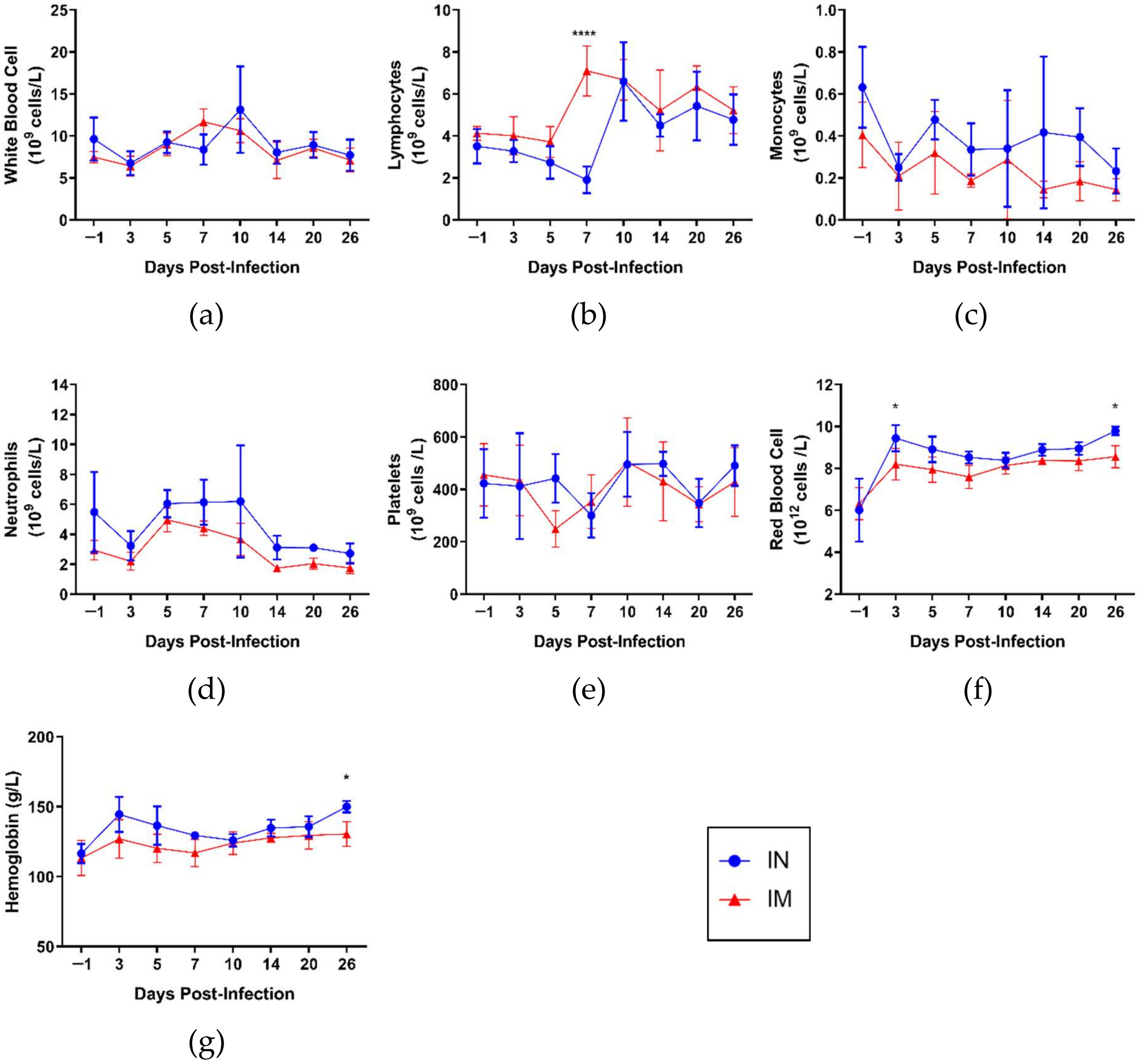

Figure S2. Average complete blood counts of TAFV-infected ferrets. Whole blood was collected from each animal on days −1, 3, 5, 7, 10, 14, 20 and 26 post infection and the concentrations of the following parameters measured: (**a**) white blood cells (WBC); (**b**) lymphocytes (LYM); (**c**) monocytes (MON); (**d**) neutrophils (NEU); (**e**) platelets (PLT); (**f**) red blood cells (RBC) and (**g**) hemoglobin (HB). IM, intramuscular inoculation; IN, intranasal inoculation.

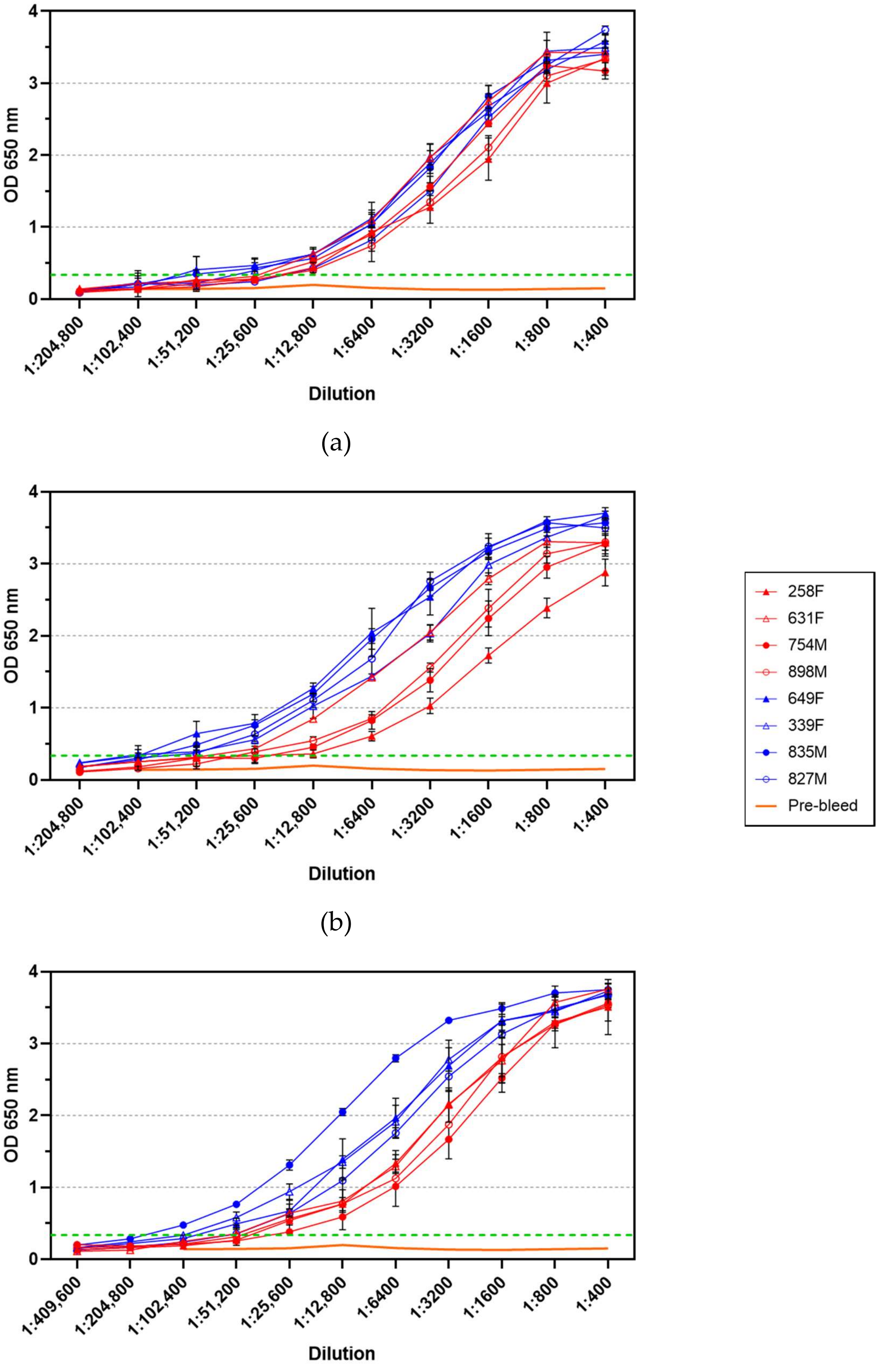

(c)

Figure S3. Detection of TAFV GP-specific IgG by ELISA. Serum samples collected from each animal on 14 (**a**), 20 (**b**) and 26 dpi (**c**) were subjected to ELISA to quantify circulating TAFV GP-specific IgG, indicated by fluorescence optical density (OD). The average of all pre-bleed (−1 dpi) samples is depicted by the orange line, and the positive cut-off is denoted by the green dotted line.

Table S1. RT-qPCR primer/probe sequences used for the detection of TAFV RNA

Target	Forward Primer (5′-3′)	Reverse Primer (5′-3′)	Probe
TAFV L	CGTCA TCGCA TTGTT GCAA	CACTC GACTG TGGGC TTCTG	6FAM-ATGAG TCCTC CCACG ATCAT GTTTG TGC-BHQ1
TAFV NP	ACCAG CACCT GTTTA TCGGA GTA	ACTCA CTTGG TTTGG TTGCT TCT	6FAM-AGAAA AGGAG CCCCT CCCCG CAAG-BHQ1

Table S2. Endpoint Titers of TAFV GP-specific IgG

**Route**	**Animal ID**	**Day Post-Infection**		
		**14**	**20**	**26**
Intramuscular (IM)	258F	1:12,800	1:25,600	1:25,600
	631F	1:12,800	1:25,600	1:51,200
	754M	1:12,800	1:12,800	1:25,600
	898M	1:12,800	1:25,600	1:12,800
Intranasal (IN)	649F	1:51,200	1:51,200	1:51,200
	339F	1:25,600	1:102,400	1:51,200
	835M	1:51,200	1:51,200	1:102,400
	827M	1:12,800	1:51,200	1:51,200

Values reported represent the lowest reciprocal dilution above the cut-off
